# “Who Are You”: development of a holistic, social-emotionally grounded curriculum framework using the Delphi technique

**DOI:** 10.3389/fpsyg.2026.1776122

**Published:** 2026-05-11

**Authors:** Melih Dikmen, Fatih Özyurt, Semih Dikmen, Emre Kilavuz, Gözde Modan, Hüseyin Kurt, Remzi Alperen Can, Tuğrul Bayindirli

**Affiliations:** 1 Faculty of Education, Firat University, Elazig, Türkiye; 2 Faculty of Engineering, Firat University, Elazig, Türkiye; 3 Independent Researcher, Istanbul, Türkiye

**Keywords:** 21st-century skills, curriculum framework, Delphi technique, holistic curriculum, social and emotional learning

## Abstract

**Introduction:**

This study aimed to develop a consensus-based holistic curriculum framework, Who Are You (WAY), intended to support learners’ cognitive, social, and emotional development from early childhood through upper secondary education.

**Methods:**

The conceptual foundation of the study was informed by holistic education perspectives, the social and emotional learning literature, CASEL’s competency framework, discussions of 21st-century skills, and the multiple intelligences perspective. A three-round Delphi design was employed. The initial item pool was generated through a multi-source process combining an extensive literature review with a needs assessment involving key educational stakeholders. In the first phase, 390 draft learning outcomes were developed across 13 themes and evaluated by an interdisciplinary expert panel recruited from the seven geographical regions of Türkiye.

**Results:**

A total of 656 experts were invited, 294 consented to participate, and 214 completed the first-round survey. Panel retention remained strong across rounds, reaching 80% in the final round. Panel quality indicators supported the credibility of the consensus process: the authority coefficient remained high throughout the study (K ≈ 0.76–0.81), and Kendall’s coefficient of concordance increased across rounds (W = 0.55 to 0.83, p < 0.001). The Delphi process resulted in a final curriculum framework comprising 312 learning outcomes organized into 84 units under 13 themes, including self-awareness and self-management, empathy and social competence, well-being and resilience, critical thinking and problem solving, culture and language, nature and ecological awareness, arts and creativity, and technology/artificial intelligence literacy.

**Discussion:**

The findings indicate that the WAY framework received broad interdisciplinary endorsement and may provide a useful basis for integrating social and emotional learning, future-oriented competencies, and holistic development goals into curriculum design. However, its practical applicability and educational value remain to be examined through future implementation studies.

## Introduction

The holistic development approach in education aims to foster individuals’ social, emotional, and academic success in a comprehensive manner (Chakraborty, 2024). Historically, pioneering educators such as John Dewey and Maria Montessori emphasized the necessity of addressing learning as an integrated process encompassing cognitive, affective, and social dimensions (Dewey, 1986; Ramaila, 2025). In a similar vein, Noddings (2003) argued that education should not be confined to the development of cognitive skills such as literacy and numeracy, but should also incorporate approaches that promote individuals’ ethical, aesthetic, physical, and emotional development. Within this perspective, education is expected to serve the development of individuals’ cognitive, affective, and psychomotor skills, thereby preparing them as active and responsible members of society (Hoque, 2016). This viewpoint underscores that education should not be limited to cognitive objectives alone; rather, students need to be equipped with lifelong, sustainable competencies by fostering social and emotional skills such as empathy, self-awareness, and self-regulation. Accordingly, the integration of social and emotional learning structures into educational and instructional settings has become increasingly important.

Research in the field of Social and Emotional Learning (SEL) has consistently highlighted the strong relationship between academic achievement, emotional intelligence, and social skills. Salovey and Mayer (1990) conceptualized emotional intelligence as the capacity to perceive, understand, and manage one’s own emotions as well as those of others, whereas Goleman (1995) framed it as a set of competencies, such as empathy, self-awareness, self-regulation, and social skills, that can be systematically developed through educational processes. Empirical evidence indicates that individuals with higher levels of emotional intelligence demonstrate more effective interpersonal communication (Raeissi et al., 2019), greater inclination toward teamwork (Moore and Mamiseishvili, 2012), and more functional coping strategies in the face of stress (Jung et al., 2019). From early childhood onward, the acquisition of skills related to recognizing emotions, empathizing with others, and regulating behavior is considered crucial for both social adjustment and academic success (Goleman, 1995; Durlak et al., 2011). Indeed, research has shown that emotional and social competencies significantly and positively predict students’ engagement in learning and academic performance (Zins et al., 2007; Dumitrescu, 2023). While positive affective experiences reinforce learning, negative emotional experiences have been associated with maladaptive behaviors such as academic procrastination (Dymnicki et al., 2013). From this perspective, SEL offers a holistic framework that extends beyond individual development to encompass the social and emotional context of learning.

Theoretical and applied research in the SEL domain is largely grounded in the emotional intelligence approach and is structured in alignment with the competency-based framework developed by the Collaborative for Academic, Social, and Emotional Learning (CASEL) (Payton et al., 2000; CASEL, 2023). While the emotional intelligence approach explains emotional functioning through psychological dimensions, the CASEL framework integrates these competencies into education systems in a structured and actionable manner. The CASEL model conceptualizes social and emotional learning across five core competency domains: self-awareness, self-management, social awareness, relationship skills, and responsible decision-making. Moreover, these domains are systematically linked to curricula, school policies, and classroom practices (Payton et al., 2000; CASEL, 2023). One of the model’s distinctive features is its explicit emphasis on responsible decision-making as a separate competency, highlighting ethical reasoning, social responsibility, and civic engagement (Jones and Kahn, 2017). In this respect, the CASEL framework does not restrict SEL to individual emotional regulation skills; instead, it conceptualizes academic achievement, social cohesion, and democratic participation as interrelated dimensions of holistic development. A substantial body of empirical research has demonstrated that schools implementing CASEL-based curriculum report increased academic achievement, reduced problem behaviors, and the emergence of more inclusive school climates (Durlak et al., 2011; CASEL, 2023).

In the contemporary century, individuals are required to possess a range of competencies in order to succeed. Reports by World Economic Forum (2025) and OECD (2019) emphasize that skills such as creativity, critical thinking, collaboration, communication, and adaptability are at least as essential as foundational academic skills in the 21st century. According to Reaves (2019), one of the primary reasons for the growing prominence of these skills is the transformation of the world of work driven by the Fourth Industrial Revolution, characterized by advancements in artificial intelligence, robotics, and automation. Consequently, a shared conclusion across numerous educational studies is that curricula should equip students with 21st-century skills such as flexibility, adaptability, observation, empathy, creativity, innovation, artificial intelligence literacy, and learning to learn (Stoller et al., 2013; Henriksen et al., 2014; Patel et al., 2019; Dai et al., 2023). In the future world, it is no longer sufficient for students to be merely endowed with existing knowledge; rather, they must be prepared with the capacity to sustain learning, adapt to novel situations, use technology effectively, and uphold ethical values (Trilling and Fadel, 2009; Voogt and Roblin, 2012). Within this context, educational curricula are expected to promote individuals’ holistic development across academic, social, and emotional domains.

Within the framework of holistic development, Gardner’s (1983) Theory of Multiple Intelligences occupies a prominent position in the educational sciences literature. Gardner (1983) proposed that intelligence is not a unidimensional construct; instead, individuals may exhibit abilities and predispositions across diverse domains, including linguistic, logical–mathematical, visual–spatial, musical–rhythmic, bodily–kinesthetic, interpersonal, intrapersonal, and naturalistic intelligences. According to this theory, schools should support students’ development not only in verbal and numerical domains but also across physical, artistic, social, and nature-related domains of ability. Indeed, contemporary research has emphasized that activities traditionally marginalized in conventional education, such as physical education, drama, music, nature-based learning, and visual arts, play an important role in fostering individuals’ diverse intelligence potentials (Tomporowski et al., 2015a, 2015b; Brovchak et al., 2024). Gardner’s (1983) approach underscores the importance of diversity and individualization in curriculum development by advancing the notion that every child possesses unique strengths. Although the theory of multiple intelligences has been subject to criticism (Eisner, 2002), numerous studies have highlighted the significance of the competencies encompassed within this framework for individuals’ holistic development (Mumtazah et al., 2025). The evidence supporting multiple intelligences primarily relies on anecdotal examples rather than comprehensive empirical data (Shearer, 2018), and factor studies have not demonstrated that the proposed intelligences are independent of one another (Ferrero et al., 2021). Despite these criticisms, researchers (Kezar, 2001) continue to emphasize how the diverse competencies described in this framework contribute meaningfully to comprehensive human development across different domains. Operationally, the MI framework suggests that a holistic curriculum should address each intelligence domain through dedicated thematic content rather than privileging verbal and numerical abilities alone (Gardner, 1983). For instance, linguistic intelligence may be cultivated through language and culture-oriented themes; logical-mathematical intelligence through thinking skills, problem solving, and scientific inquiry; musical-rhythmic and bodily-kinesthetic intelligences through arts, creativity, and physical development; interpersonal and intrapersonal intelligences through social–emotional and self-awareness content; and naturalistic intelligence through ecological and nature-based learning experiences. In this respect, each of Gardner (1983) intelligence domains can find a corresponding curricular theme within a holistic framework, ensuring that the full spectrum of human potential is systematically addressed. Accordingly, there is a clear need for curricula that support the development of individuals’ physical, artistic, social, and nature-related abilities in an integrated manner.

Beyond social and emotional competencies, contemporary curricula are increasingly expected to include developmental components that support learners’ emerging sense of self, personal meaning, and identity formation. Identity development theory constitutes another important foundation for understanding why contemporary curricula should address the development of the self in a more systematic and developmentally responsive manner Erikson (1968) psychosocial development framework established that identity formation is not confined to adolescence but unfolds as an ongoing, stage-sensitive process across the lifespan, with each developmental stage presenting characteristic psychosocial challenges that shape the individual’s sense of self. Building on this foundation, Marcia (1980) operationalized identity formation through the concepts of exploration and commitment, identifying four identity statuses, diffusion, foreclosure, moratorium, and achievement, that illuminate how individuals negotiate between established and emergent self-definitions. More recently, Luyckx et al. (2006) extended this model to encompass the dual processes of identity formation and identity evaluation, underscoring the dynamic and recursive nature of self-construction. From a self-concept perspective, Harter (1999) demonstrated that children’s self-concept differentiates across domains, academic, social, physical, and global, from middle childhood onward, and that a coherent, positively valenced self-concept is significantly associated with well-being, motivation, and academic engagement. These theoretical insights converge on a shared conclusion: that supporting identity development within educational curricula is not merely a philosophical aspiration but a developmentally justified and empirically grounded imperative. It is within this framework that a holistic curriculum may most meaningfully position identity construction not as a peripheral theme but as an organizing thread running through its thematic domains, providing learners with structured opportunities to explore who they are, what they value, and how they relate to others and the world.

### The present study

The theoretical framework outlined above suggests the need for a broader perspective in curriculum design. In both Türkiye and the wider international context, contemporary education systems increasingly call for curriculum frameworks that integrate social and emotional learning with traditional academic content (OECD, 2018). In response to this need, the present study sought to develop the WAY framework through the Delphi technique. WAY is conceptualized as a holistic curriculum framework intended to support learners’ cognitive, affective, and social development in an integrated manner, from early childhood through the end of secondary education. Its content encompasses a broad range of learning outcomes, extending from self-awareness and self-management to social awareness and community-related competencies; from critical thinking and responsible decision-making to creative expression and storytelling; and from ecological awareness to technological literacy and awareness of artificial intelligence. In this sense, the WAY framework is intended to support learners’ development across multiple interrelated domains that are increasingly regarded as important in contemporary education.

In addition, the WAY framework incorporates identity development as an important dimension within a broader holistic structure by emphasizing self-awareness, meaning-making, personal strengths, and self-understanding. It is intended to help learners recognize and interpret their emotions, understand their personal boundaries and needs, and reflect on their strengths and potentials as part of ongoing self-development. By emphasizing intrinsic motivation, sense of purpose, self-compassion, and self-respect, the framework approaches identity development as a dynamic and evolving process rather than as a fixed outcome. In this way, WAY is intended to contribute to well-being, resilience, and lifelong learning through a holistic educational approach in which identity development is treated as one important dimension among others.

Accordingly, given the growing need for curricula that integrate social–emotional, cognitive, creative, and future-oriented competencies, the present study aimed to develop a consensus-based holistic curriculum framework entitled WAY through the Delphi technique.

## Method

### Research design

In the present study, the Delphi technique was employed to achieve expert consensus during the development of the WAY curriculum. Delphi is a structured, iterative technique conducted through successive rounds with the participation of experts and is grounded in the principle of anonymity (Jorm, 2025). The process advances through controlled feedback provided at the end of each round. Fundamentally, the Delphi technique involves the systematic collection and comparison of expert judgments and aims to establish consensus on a given issue (Bahar and Somuncu Demir, 2021). Within the Delphi process, experts respond to the same set of questions across multiple rounds. At the conclusion of each round, anonymized summaries of group responses and synthesized feedback from the previous round are compiled and redistributed to the experts (Boulkedid et al., 2011). This procedure enables experts to review their own judgments in light of the collective perspectives of the panel. As a result, discrepancies among individual responses tend to diminish over successive rounds, facilitating convergence toward a shared consensus. The process continues until a predetermined number of rounds is completed, a targeted level of consensus is achieved, or expert responses reach a stable equilibrium (Chuenjitwongsa et al., 2017). This methodological structure of the Delphi technique minimizes the influence of dominant viewpoints that may emerge in face-to-face group discussions, thereby enabling a more balanced and reliable consensus-building process (Boulkedid et al., 2011).

In the present study, a three-round Delphi design was adopted to allow iterative reconsideration of expert judgments while minimizing panel fatigue and attrition. Although there is no universally fixed standard regarding the optimal number of Delphi rounds, three rounds are widely considered sufficient to achieve refinement and stabilization of expert opinion without imposing excessive response burden on participants (Jorm, 2025). This design was considered particularly appropriate for the present study because the WAY curriculum addressed a broad and interdisciplinary thematic structure requiring both initial exploration and subsequent refinement of judgments across rounds. In addition, the Delphi process was planned to continue until either the targeted consensus criteria were met or the ratings demonstrated clear stability across successive rounds.

### Research team

The research team of the present study consisted of three researchers holding doctoral degrees in curriculum and instruction and computer engineering, respectively, along with four researchers who conduct academic work in the fields of social and emotional development. In addition, an educational technology specialist with expertise in future-oriented skills education contributed to the study. The research team collaboratively carried out all stages of the Delphi process, including the development of Delphi questionnaires, the invitation and coordination of the expert panel, the analysis of data obtained from each round, and the preparation of feedback reports for participating experts. Decisions at each stage of the curriculum development process were made through team-based discussion and consensus. Determinations regarding which items should be revised, added, or removed across Delphi rounds were evaluated by the research team by jointly considering quantitative results from the expert panel and qualitative feedback provided by the experts. Qualitative feedback was reviewed through a structured decision process. Suggestions were retained when they (a) clarified ambiguous wording, (b) reduced redundancy, (c) improved developmental or conceptual fit, or (d) identified content gaps consistent with the broader goals of the curriculum. Suggestions were not adopted when they were overly narrow, context-specific, duplicative of existing items, or conceptually inconsistent with the intended scope of the WAY framework. In cases of disagreement, final decisions were made through team discussion by jointly considering the quantitative ratings, thematic coherence of the curriculum, and the explanatory quality of experts’ written comments.

## Development of the expert questionnaire

### Literature review

A comprehensive literature review was conducted to generate the questionnaire items to be presented to the expert panel. To ensure access to both international and national sources, studies indexed in the Web of Science, Scopus, ERIC, PsycINFO, TRDİZİN, and Google Scholar databases were examined (Spirito et al., 1988; Adams, 1989; Scheffler, 1992; Hargreaves, 1995; Alexander and Luckman, 2001; Graham-Jolly, 2003; Parsons, 2004; Broderick and Metz, 2009; Soutter et al., 2012; Jones and Wyse, 2013; Stoller et al., 2013; Henriksen et al., 2014; Patel et al., 2019; Dai et al., 2023; Dilekçi and Karatay, 2023). Within the scope of the review, the following keywords were used in Turkish and English: science, awareness, well-being, thinking skills, sport, culture, philosophy, quantum, emotional intelligence, empathy, nature, art, artificial intelligence, creativity, 21st-century skills, identity development, self-concept, and curriculum development. The reviewed literature encompassed previous research, existing curricula, and scale development studies related to the core skills and competency domains targeted by the WAY curriculum. Findings from the literature review served as the primary foundation for generating the initial items of the Delphi questionnaire and were also considered instrumental in supporting the content validity of the study. To prevent the literature-derived framework from excessively constraining expert input, open-ended response sections were incorporated into the questionnaire, allowing panelists to propose new suggestions and perspectives. This approach is considered to enhance the reliability of the Delphi process (Chuenjitwongsa et al., 2017). In addition to the literature review, data were collected from field stakeholders to identify context-specific needs relevant to the educational settings in which the program is intended to be implemented. For this purpose, open-ended questionnaires and brief online forms were administered to a sample consisting of teachers, school administrators, students, and parents. A total of 17 parents, 9 school administrators, 20 students, and 20 teachers participated in this phase. Through these instruments, participants’ expectations regarding individual, social, emotional, and academic development, challenges encountered in educational environments, and perceived needs related to program content were identified. The qualitative data obtained were analyzed using content analysis, and the resulting themes were evaluated in conjunction with findings from the literature. This multi-source approach enabled the initial Delphi items to be grounded in both theoretical foundations and field-specific realities (Jorm, 2025), thereby strengthening content validity during the item development process.

The qualitative data collected from field stakeholders were integrated into the item development process through a structured thematic synthesis procedure. Responses from teachers, students, parents, and school administrators were independently coded by two researchers using NVivo (Version 14), yielding a set of recurring themes organized around perceived developmental needs, contextual challenges, and content expectations. These themes were then mapped onto the 13 predefined curriculum domains and cross-referenced with the literature-derived item pool. Where stakeholder data confirmed existing literature-based items, no additions were made. Where novel themes emerged that were not adequately represented in the literature (e.g., students’ expressed need for identity affirmation practices and teachers’ concerns about emotional regulation in technology-mediated environments), new draft items were generated. A total of 48 items in the initial pool of 390 were directly attributable to stakeholder-derived themes, with the remainder grounded in the literature review. This dual-source triangulation strengthened the ecological validity of the initial item pool and ensured that the curriculum’s conceptual framework was anchored in both theoretical knowledge and lived educational experience.

### Item pool

Based on the literature review and the needs analysis data obtained from field stakeholders, the core thematic domains targeted by the WAY curriculum were identified. The principal thematic areas proposed within the program are as follows:

- Self-Awareness and Identity
- Emotional Intelligence and Empathy
- Future Readiness
- Well-Being and Mindfulness
- Thinking Skills and Problem Solving
- Physical Development and Movement
- Language and Culture
- Community and Collaboration
- Philosophy
- Nature, Agriculture, and Ecological Awareness
- Arts and Creativity
- Technology and Artificial Intelligence
- Science and Quantum Concepts

For each thematic domain, the research team identified relevant subdimensions and skills and developed draft items (learning outcomes) based on data derived from both the literature and the field study. Each item was formulated as clearly and explicitly as possible to facilitate expert evaluation regarding whether the corresponding skill should be included in the program content and the degree of emphasis it should receive. In drafting the items, prior curriculum development studies related to these thematic areas were consulted (Alexander and Luckman, 2001; Graham-Jolly, 2003; Parsons, 2004; Broderick and Metz, 2009; Soutter et al., 2012; Jones and Wyse, 2013; Stoller et al., 2013; Henriksen et al., 2014; Patel et al., 2019; Dai et al., 2023; Dilekçi and Karatay, 2023). At the same time, necessary adaptations and additions were made by taking into account the unique context and specific needs of the WAY curriculum. Initially, 30 items were generated for each thematic domain, resulting in a total of 390 draft items. The decision to begin with a relatively large item pool was intentional. Given the comprehensive and interdisciplinary nature of the WAY curriculum, it was considered methodologically important to represent each thematic domain broadly at the initial stage rather than prematurely narrowing the scope of the curriculum. In Delphi-based curriculum development, an initially inclusive item pool allows experts to eliminate, refine, and prioritize outcomes through successive rounds, thereby supporting both conceptual breadth and systematic content refinement (Jorm, 2025). Accordingly, the 390-item structure was designed as an exploratory starting framework to be progressively reduced and sharpened through expert consensus. This item pool was pilot-tested prior to being presented to the expert panel in the first round of the Delphi study. Subsequently, two academics specializing in linguistics reviewed the items to assess their clarity and comprehensibility, leading to revisions of 73 items that were identified as difficult to understand. As a result, the finalized set of items constituted a preliminary questionnaire representing the core content components of the WAY curriculum and was deemed ready for expert evaluation. In addition, an introductory section was included in the questionnaire to provide instructions for participating experts and to collect demographic information, including gender, age, educational background, academic title, field of expertise, and professional experience.

### Selection of the expert panel

The selection of the expert panel constitutes an important component in supporting the credibility and content relevance of findings derived from the Delphi technique (Chuenjitwongsa et al., 2017). Accordingly, panel recruitment was guided by predefined qualitative criteria. The primary selection criterion was that experts possess substantial knowledge and professional experience relevant to the thematic domains addressed by the WAY curriculum. Potential experts were selected through purposive sampling from two main groups: (a) academics with disciplinary expertise relevant to the WAY themes and at least 5 years of professional experience, and (b) experienced practitioners occupying senior or specialist roles in fields directly related to the curriculum domains.

To strengthen thematic coverage, expert recruitment was organized around the conceptual architecture of the WAY curriculum rather than on the basis of general educational expertise alone. Specifically, panelists from educational sciences, psychology, and counseling were recruited to evaluate outcomes in the Self-Awareness and Identity, Emotional Intelligence and Empathy, Well-Being and Mindfulness, and Community and Collaboration domains. Experts from philosophy and sociology contributed primarily to the Philosophy, Language and Culture, and Community and Collaboration themes. Scientists and engineers (e.g., physics, environmental engineering, agricultural engineering, and biology) evaluated items in the Nature, Agriculture, and Ecological Awareness and Science and Quantum Concepts themes. Computer engineers and STEM educators provided specialist input on the Technology and Artificial Intelligence theme. Visual arts educators and drama instructors evaluated the Arts and Creativity domain, while sports scientists and physiotherapists contributed to the Physical Development and Movement theme. Although all panelists were invited to evaluate all items in order to preserve the holistic character of the process, this domain-based alignment was intended to ensure that each thematic cluster received input from multiple specialists whose expertise was directly relevant to its content.

Initial contact with potential panelists was established via email, through which a formal invitation outlining the purpose of the study and the procedures of the Delphi process was sent. To increase contextual breadth within the Turkish educational setting, 586 experts from the seven geographical regions of Türkiye were initially invited to participate. Of these, 254 indicated their willingness to participate and provided digital informed consent. In the first round, 191 of these experts actively participated. Because some thematic areas were initially represented by fewer experts than anticipated, an additional 70 specialists from the relevant fields were invited. Forty accepted the invitation, and 23 participated in the first round. It was therefore concluded that a sufficient level of thematic coverage had been achieved across the curriculum domains to proceed with the Delphi process. In total, 656 experts were invited, 294 consented to participate, and 214 completed the first-round survey.

The interdisciplinary composition of the panel was regarded as a methodological strength because the WAY curriculum spans multiple developmental and knowledge domains. At the same time, the research team recognized that panel heterogeneity could reduce the likelihood of rapid convergence, particularly in the early rounds. In addition, because some domains may have been supported by more densely represented disciplines than others, consensus patterns were interpreted cautiously in relation to the thematic breadth of the panel. For this reason, panelists were permitted to indicate “Unable to comment / Insufficient knowledge” when appropriate, and convergence was interpreted as developing progressively across rounds rather than being expected at the outset.

Prior to data collection, ethical approval for the study was obtained from the Social and Human Sciences Research Ethics Committee of Fırat University (Approval No. E-50716828-100-755886). Digital informed consent was obtained from all Delphi participants before their inclusion in the study. For the stakeholder needs-analysis phase, informed consent was obtained from adult participants, including teachers, school administrators, and parents. For student participants, parental permission was obtained and age-appropriate assent procedures were followed in accordance with the ethical requirements of the study. The demographic characteristics of the participating experts are presented in Table 1.

**Table 1:** Demographic characteristics of the experts (*n* = 214).

Category	Variables	n	%
Gender	Male	119	55.61
	Female	95	44.39
Age group (years)	30–40	135	63.08
	41–50	63	29.44
	>50	16	7.48
Education level	Bachelor’s degree	32	14.95
	Master’s degree	53	24.77
	Doctoral degree (PhD)	129	60.28
Academic title/position	Specialist	33	15.42
	Research Assistant	52	24.30
	Research Assistant (PhD)	67	31.31
	Lecturer	1	0.47
	Lecturer (PhD)	16	7.48
	Assistant Professor	28	13.08
	Associate Professor	12	5.61
	Professor	5	2.34
Professional experience	5–10 years	68	31.78
	11–15 years	97	45.33
	16–20 years	43	20.09
	>20 years	6	2.80
Field of expertise	Educational sciences	26	12.15
	Physics	15	7.01
	Psychology	16	7.48
	Sports sciences	13	6.07
	Sociology	18	8.41
	Communication sciences	15	7.01
	Philosophy	9	4.21
	Visual Arts	9	4.21
	Language education	11	5.14
	Anthropology	10	4.67
	Environmental engineering	15	7.01
	Agricultural engineering	8	3.74
	Computer and software engineering	16	7.48
	Psychological counselor	7	3.27
	Drama instructor	5	2.34
	STEM educator	3	1.40
	Public health specialist	4	1.87
	Physiotherapist	6	2.80
	Biologist	8	3.74

As shown in Table 1, 55.61% of the expert panelists participating in the study were male (*n* = 119), while 44.39% were female (*n* = 95). An examination of the age distribution indicates that the majority of panelists were between 30 and 40 years of age (63.08%), followed by those aged 41–50 years (29.44%), with experts over the age of 50 constituting a relatively smaller proportion of the panel (7.48%). Regarding educational attainment, the panel was predominantly composed of individuals with postgraduate qualifications. Specifically, 60.28% of the participants held a doctoral degree, 24.77% had a master’s degree, and 14.95% had completed a bachelor’s degree. In terms of academic rank, the panel demonstrated a balanced composition, including early-career academics such as specialists (15.42%), research assistants (24.30%), research assistants with a doctoral degree (31.31%), and lecturers with a doctoral degree (7.48%), alongside mid- and senior-career academics holding the titles of assistant professor (13.08%), associate professor (5.61%), and professor (2.34%). With respect to professional experience, a substantial proportion of the panelists had between 11 and 15 years (45.33%) or 5–10 years (31.78%) of experience, while 20.09% reported 16–20 years and 2.80% reported more than 20 years of professional experience. This distribution suggests that the panel comprised experts who combined up-to-date practical knowledge with substantial professional expertise. The distribution of fields of expertise reflects the interdisciplinary nature of the WAY curriculum. The panel included experts primarily from educational sciences (12.15%), as well as psychology (7.48%), physics (7.01%), sports sciences (6.07%), sociology (8.41%), communication sciences (7.01%), philosophy (4.21%), visual arts (4.21%), language education (5.14%), anthropology (4.67%), environmental engineering (7.01%), agricultural engineering (3.74%), and computer and software engineering (7.48%). In addition, the inclusion of professionals from practice-oriented fields, such as psychological counseling, drama education, STEM education, public health, physiotherapy, and biology, further supported the holistic consideration of the program’s social–emotional, artistic, physical, technological, and scientific dimensions. This broad and well-balanced distribution of expertise ensured that all thematic domains of the WAY curriculum, including self-awareness, social–emotional development, creativity, nature and agriculture, technology, and science, were adequately represented within the Delphi panel, thereby strengthening the content validity of the consensus-based findings.

### Questionnaire administration process

The Delphi process was conducted through the administration of the expert questionnaire to the same panel across multiple rounds. The process comprised three rounds. This structure was selected to balance methodological rigor with feasibility. The first round was designed to obtain broad expert evaluations and qualitative suggestions regarding the initial item pool, the second round to allow reconsideration of items in light of aggregated group feedback, and the third round to resolve remaining ambiguity and determine whether judgments had stabilized. Thus, the three-round structure was considered sufficient to support refinement, convergence, and final decision-making without creating unnecessary repetition for panelists. As the Delphi technique aims to achieve consensus among a group of experts on a given topic, it is expected that all panelists evaluate the items presented, even in interdisciplinary contexts. When the number of items is extensive, grouping items under thematic headings may facilitate clarity and comprehensibility (Jorm, 2025). Accordingly, in the first round, panelists were presented with a total of 390 items-30 items under each of 13 predefined thematic domains, via an online questionnaire. Experts were asked to evaluate each item in terms of its relevance and importance for the WAY curriculum. Evaluations were conducted using a five-point Likert scale (1 = *Not appropriate at all*, 5 = *Highly appropriate*). Given the interdisciplinary nature of the WAY curriculum, panelists were informed that they could select the option “Unable to comment / Insufficient knowledge” if they felt unable to evaluate a particular item. Although this option was provided, experts were encouraged to assess all items. In addition, a comment box was included for each item, allowing panelists to provide qualitative feedback, suggestions for improvement, or justifications alongside their numerical ratings. At the end of the first round, all responses were compiled and analyzed by the research team. Special attention was paid to comments accompanying low-frequency or minority positions. Even when such views did not reflect the dominant panel tendency, they were reviewed to determine whether they identified conceptual blind spots, wording ambiguities, or context-specific considerations. Thus, minority feedback was not treated as irrelevant by default; rather, it was examined as a potential source of refinement, particularly during item revision and item-addition decisions. For each item, the number of responding experts, mean score, median, range, interquartile deviation, standard deviation, and score distribution were calculated. Furthermore, the percentage of agreement was computed by determining the proportion of panelists who rated each item positively. Response patterns were monitored at the end of the first round, and reminder messages were sent to non-responding participants when necessary to maintain a high response rate. In the second round, items that did not reach consensus in the first round, along with newly added items derived from panelists’ suggestions, were re-evaluated. Panelists received the questionnaire link together with a summary of the first-round results. During this round, experts were provided with the group’s mean, median, range, interquartile deviation, standard deviation, number of respondents, and their own previous rating for each item. Panelists were asked to reconsider and re-rate the items by taking into account the overall tendency of the group, either revising or maintaining their initial judgments as they deemed appropriate. Following the second round, the data were reanalyzed and updated statistics for each item were generated. Items showing substantial convergence were identified, while those still lacking consensus were deemed appropriate for a third round. In the third round, only items that remained ambiguous or elicited divergent opinions after the second round were included. Panelists conducted a final evaluation by considering the cumulative feedback and statistical summaries. At the conclusion of the third round, the Delphi process was terminated because no substantial changes were observed in item ratings, the dispersion of responses had decreased, and expert judgments had largely stabilized, indicating that an additional round was unlikely to yield meaningful changes in the overall pattern of consensus (Chuenjitwongsa et al., 2017).

### Data analysis

Data obtained from the Delphi study were analyzed using quantitative statistical methods. All statistical analyses were conducted using SPSS version 22. After each round, the collected data were initially examined using descriptive statistics. For each item, measures such as the mean and standard deviation were calculated. Various criteria for determining consensus levels are reported in the Delphi literature. Because Delphi studies do not rely on a single universally accepted consensus threshold, the present study adopted a combined approach that was considered both methodologically rigorous and practically interpretable for a large, interdisciplinary panel. A 75% agreement threshold was selected as a relatively stringent criterion to indicate substantial endorsement of an item while remaining realistic for a curriculum framework spanning diverse thematic domains. In addition to percentage agreement at the item level, Kendall’s coefficient of concordance (Kendall’s W) was used to assess the overall degree of convergence across expert ratings within each round. The combined use of these indicators allowed consensus to be evaluated both locally, at the level of individual items, and globally, at the level of panel-wide agreement. In line with this rationale, percentage agreement and Kendall’s W were interpreted jointly in order to obtain a more robust picture of consensus formation across the Delphi rounds. In terms of percentage agreement, an item was accepted by panel consensus if at least 75% of the experts rated it as *appropriate* (Likert scale values of 4 or 5). Similarly, an item was rejected if at least 75% of the panelists rated it as *not appropriate* (Likert scale values of 1 or 2). These threshold values are consistent with commonly accepted consensus criteria in Delphi research (Jorm, 2025). To quantitatively express the overall level of agreement within the panel, Kendall’s coefficient of concordance (Kendall’s W) was calculated at the end of each round (Grzegorzewski, 2006). Kendall’s W ranges from 0 to 1, indicating the degree of agreement among experts’ rankings or ratings. A value of 0 reflects no agreement within the panel, whereas a value of 1 indicates complete consensus. The literature suggests that W values above 0.50 indicate strong agreement, while values of 0.70 or higher represent very high levels of consensus (Sun et al., 2024). In the present study, the statistical significance of Kendall’s W coefficients obtained after each round was tested using the chi-square test. Statistically significant W values indicate that expert ratings were not randomly distributed and that a meaningful level of agreement was achieved (Martínez-Sánchez, 2021). Finally, to assess the overall level of panel expertise and to ensure panel quality, the expert authority coefficient described in the literature was calculated. The expert authority coefficient is a composite indicator that quantitatively reflects each panelist’s level of expertise and the reliability of their judgments (Martínez-Sánchez, 2021). This coefficient is derived from two components: experts’ self-assessed level of knowledge and the extent to which they justify their judgments. The expert authority coefficient (K) was computed as the arithmetic mean of two components: the expert familiarity coefficient (Cs) and the judgment basis coefficient (Ca), using the formula K = (Cs + Ca) / 2. Cs reflected self-rated familiarity with the relevant thematic area on a 0–1 scale, whereas Ca reflected the extent to which the expert justified their ratings by drawing on professional experience, theoretical knowledge, empirical evidence, or contextual knowledge (Dalkey and Helmer, 1963; Martínez-Sánchez, 2021). For instance, drawing on theoretical knowledge contributed a weight of 0.3, while professional experience contributed 0.5, and other sources were assigned lower weights.

In addition, the coefficient of variation (CV) was calculated as a supplementary indicator of response dispersion and consensus. CV was computed by dividing the standard deviation by the mean for the relevant set of ratings (CV = SD / M), with lower values indicating lower variability and greater agreement among experts. In the present study, CV values were used as a descriptive indicator rather than as a primary criterion for item retention. After completion of the Delphi rounds, average CV values were also calculated at the thematic-unit level for the final set of retained learning outcomes in order to summarize the consistency of expert judgments within each thematic cluster. Consistent with the literature, CV values below 0.25 were interpreted as indicating strong consensus, whereas lower values reflected progressively greater consistency in expert ratings.

Qualitative data obtained from field stakeholders were analyzed using thematic content analysis. All interview transcripts and written stakeholder feedback were transcribed verbatim and analyzed using NVivo (Version 14) qualitative data analysis software. An inductive coding strategy was employed, progressing from open coding to the development of categories and overarching themes (Braun and Clarke, 2006). To ensure analytical rigor and coding consistency, the qualitative data were independently coded by two researchers. Inter-coder reliability was calculated using the Miles and Huberman (1994) agreement formula (Reliability = Agreements/[Agreements + Disagreements]). In the present study, the inter-coder agreement coefficient was calculated as 0.86, indicating a high level of coding consistency and methodological reliability, exceeding the commonly accepted threshold of 0.70 in qualitative research. The resulting themes were conceptually aligned with the theoretical dimensions of the WAY curriculum and were used to contextualize and support the quantitative Delphi findings, thereby strengthening the study’s interpretive validity through methodological triangulation (Creswell and Plano Clark, 2018).

Qualitative feedback from Delphi panelists was analyzed at the item level through a structured review of recurring comments, suggested wording changes, justifications for low ratings, and proposals for additional content. These comments were not subjected to a separate full-scale thematic analysis comparable to the stakeholder dataset; rather, they were used analytically to support item-level decisions concerning revision, retention, addition, or removal. Minority-supported items were not treated uniformly. When low-support items were accompanied by comments indicating conceptual redundancy, limited relevance, or poor developmental fit, they could be removed after the first round. However, when minority feedback pointed to conceptual blind spots, wording ambiguities, or context-specific concerns, the relevant items were revised and reconsidered in subsequent rounds. In some cases, such feedback also informed the addition of new items. In this sense, qualitative panel feedback functioned as an interpretive complement to the quantitative consensus indicators.

## Results

### Expert participation

Across the three-round Delphi process, expert panel participation remained consistently high. In the first round, 214 of the 294 experts who accepted the invitation completed the questionnaire, yielding a participation rate of 72.8%. In the second and third rounds, 198 and 173 panelists, respectively, continued to participate in the evaluations. This corresponds to an approximately 80% panelist retention rate from the first to the final round. The achieved participation rate exceeds the minimum threshold of 70% commonly recommended to ensure methodological rigor in Delphi studies. In addition, more than 40% of the panelists provided written feedback for each item during the first round. Although the volume of qualitative feedback decreased in subsequent rounds, all experts remained actively engaged throughout the process and contributed to the refinement of the WAY curriculum until the final round. This high level of participation and commitment is considered critical for the reliability of panel findings and the robustness of the resulting consensus (Jorm, 2025).

### Panel authority (K) and coordination coefficient (Kendall’s W)

The level of expertise among panelists and the degree of consensus achieved during the Delphi process were examined statistically. The panel authority coefficient (K), a composite index reflecting experts’ level of expertise and the substantiation of their judgments, was calculated for all panelists. The authority coefficient exceeded 0.70 for all experts (mean K ≈ 0.79, SD = 0.05). In the literature, K values of 0.70 or higher are interpreted as indicating high reliability of expert judgments (Wang et al., 2025). In the present study, the panel’s mean K value of 0.79, with no expert scoring below the 0.70 threshold, demonstrates a high overall level of expertise among the panelists. Similarly, the degree of agreement among experts was assessed using Kendall’s coefficient of concordance (Kendall’s W), calculated at the end of each Delphi round. Kendall’s W reflects the extent to which panelists’ ratings of the same items are consistent with one another and ranges from 0 (no agreement) to 1 (complete agreement). Values of W ≥ 0.70 are generally interpreted as indicating very high consensus (Legendre, 2005). The authority and coordination coefficients for each Delphi round are presented in Table 2.

**Table 2:** Panel authority coefficient (K) and coordination coefficient (Kendall’s W) across Delphi rounds.

Round	Authority coefficient (K)	Coordination coefficient (W)	*p* value	Level of consensus
Round 1	0.76	0.55	< 0.001	Moderate consensus
Round 2	0.79	0.67	< 0.001	High consensus
Round 3	0.81	0.83	< 0.001	Very high consensus

K ≥ 0.70 indicates high expert authority; W ≥ 0.70 indicates very high consensus.

As shown in Table 2, the panel authority coefficient remained consistently high across all three rounds (K₁ = 0.76; K₂ = 0.79; K₃ = 0.81), indicating a strong level of subject-matter expertise and reliability in expert ratings. Moreover, the progressive increase in the coordination coefficient (from W₁ = 0.55 to W₃ = 0.83) demonstrates a clear trend toward greater consensus among panelists over successive rounds. The steady rise in Kendall’s W throughout the Delphi process suggests a systematic reduction in divergence of expert opinions and the formation of a shared judgment. The final-round value of W = 0.83 exceeds the threshold commonly accepted in the literature for very high consensus (W ≥ 0.70). Collectively, these findings indicate that the WAY curriculum was broadly endorsed through a strong consensus among experts from diverse disciplinary backgrounds.

### Round 1: item elimination and revisions

In the first round of the Delphi study, a total of 390 items constituting the draft content of the WAY curriculum were presented to the expert panel under 13 thematic domains. These items represented learning outcomes and skills aligned with the thirteen core themes identified by the research team based on the literature review, field data, and preliminary preparatory work. Based on the first-round voting results and expert comments, a comprehensive revision of the 390-item list was undertaken. Consensus could not be achieved for 156 items (40%) in the first round. However, lack of first-round consensus did not automatically result in item removal. Instead, these items were screened for one of three actions: revision, elimination, or reconsideration in light of qualitative feedback. Of these 156 items, 26 were removed, whereas 130 were retained in revised form for the next round. Item exclusion was guided by predefined inclusion criteria, namely a mean score below 4.0 and/or fewer than 75% of experts rating the item as appropriate. Items were removed when low quantitative support was accompanied by one or more of the following qualitative conditions: narrow contextual relevance, redundancy with stronger items, conceptual misfit with the intended scope of the curriculum, or insufficient developmental appropriateness. By contrast, items were revised and retained when experts’ comments suggested that the problem lay primarily in wording, specificity, overlap, or framing rather than in the underlying competency itself. For example, an item within the *Nature and Agriculture* domain focusing on *farm animal husbandry*, which required a highly specific contextual setting, failed to reach the required importance threshold and was therefore removed from the list. Overall, 26 items for which consensus could not be established were eliminated from the program content on the grounds that they did not adequately reflect the core scope of the program or were not considered a priority for the target population. As these items were dispersed across multiple themes, their removal did not result in substantial structural changes to the thematic framework. In addition to item elimination, wording and scope revisions were implemented during the first round. Based on qualitative feedback from the panel, 130 items were identified as requiring clearer or more inclusive phrasing. Accordingly, minor revisions and clarifications were made to nearly half of the items, guided by expert suggestions. Such refinements enhanced the distinctiveness of the items and facilitated their consistent interpretation across panelists (Jorm, 2025). Moreover, qualitative feedback in the first round generated suggestions for new items. Panelists highlighted certain content areas they perceived as underrepresented and proposed additional items for inclusion. Following an analysis of these suggestions, the research team added 19 new items to the questionnaire prior to the second round. These newly added items were intended to address content gaps identified by the panel. For instance, in response to expert recommendations emphasizing the importance of resilience within the context of social–emotional development, the item “Students should be supported in developing emotional resilience when facing challenges, changes, and stressful situations” was incorporated into the list. The eliminations and revisions conducted in the first round enabled the Delphi panel to focus on priority areas, resulting in a more concise and refined item pool for subsequent rounds.

### Rounds 2 and 3: revisions and final consensus

In the second round of the Delphi study, a revised list of 383 items across the 13 thematic domains was re-presented to the panelists. Prior to the second round, panelists were provided with summary information from the first round, including the number of responding experts, group mean, median, range, percentage agreement for each item, and anonymized expert comments. This feedback allowed experts to reconsider their judgments in light of group-level trends (Jorm, 2025). The second-round voting results indicated a substantial increase in consensus among panelists. Compared to the first round, 276 items received higher mean ratings and lower standard deviations, reflecting greater agreement. By the end of the second round, the majority of items met the predefined consensus criteria, and the level of agreement among panelists strengthened considerably (W = 0.68), indicating a statistically meaningful convergence of opinions. Nevertheless, 107 items remained without consensus at the conclusion of the second round. These items, which the panel either could not agree upon or did not rate as sufficiently important, were reviewed by the research team. Based on mean scores below 4.0, 11 items were removed from the content. For example, within the *Nature and Agriculture* theme, an item stating “Students should be supported in developing awareness of the care and basic needs of farm animals (nutrition, shelter, health, and welfare)” was excluded from the core curriculum due to persistently low ratings and its reliance on specific contextual conditions. No new items were added during the second round; instead, the focus was placed on determining the acceptance or rejection of existing items. However, the wording of 96 items that still lacked consensus was revised based on panel feedback. In the third round, the 96 items that remained ambiguous or lacked consensus after the second round were submitted for final evaluation. As panelists were provided with both the group mean from the previous round and their own prior ratings for each item, they were able to re-examine divergent viewpoints more critically. The third-round results clearly demonstrated that the panel had reached a final consensus. Of the items evaluated in this round, 36 met the predefined inclusion criteria, while 60 items from the post–second-round list were eliminated. By the final round, disagreements among panelists had been almost entirely resolved (W = 0.83). At the conclusion of the Delphi process, a consensual content framework consisting of 312 items was finalized. To improve transparency regarding the refinement of the item pool across the Delphi rounds, Table 3 summarizes the number of items retained, revised, added, and removed at each stage of the process.

**Table 3:** Flow of items across Delphi rounds.

Stage	Starting n	Items reaching consensus	Revised for reconsideration	Added	Removed	Retained after round
Initial draft	390	–	–	–	–	390
After Round 1	390	234	130	19	26	383
After Round 2	383	276	96	–	11	372
After Round 3	96*	36	–	–	60	36
Final framework	–	–	–	–	–	312

In Round 3, only the 96 items for which consensus had not yet been achieved were re-evaluated. By the end of Round 2, 276 items had already been retained. The final framework consisted of these 276 items plus 36 additional items retained in Round 3, resulting in a total of 312 learning outcomes.

As shown in Table 3, the refinement of the WAY curriculum followed a stepwise and iterative pattern across the three Delphi rounds. In the first round, 390 draft items were evaluated by the expert panel. Of these, 234 reached consensus, whereas 156 did not. Among the 156 unresolved items, 130 were revised for reconsideration and 26 were removed. In addition, 19 new items were generated on the basis of expert feedback, resulting in 383 items entering the second round. In Round 2, consensus was achieved for 276 items, while 107 items remained unresolved and were subjected to further evaluation. Of these, 96 were revised and retained for the third round, whereas 11 were removed. In Round 3, only the 96 revised items from the second round were re-evaluated by the expert panel. At this stage, 36 items reached consensus and 60 were removed. Taken together, this process resulted in a final framework consisting of 312 learning outcomes. These findings indicate that items not reaching consensus in a given round were not handled through a single rule; rather, they were reviewed in light of both quantitative ratings and qualitative expert feedback and were subsequently revised, retained, added, or removed accordingly.

### Evaluation of program items

As presented in Table 4, the 312 items structured under the 13 thematic domains of the WAY curriculum were organized into a total of 84 thematic units. Following the completion of the Delphi process, the items that achieved consensus were reorganized under a thematic structure by taking into account content similarities and shared learning objectives. At this stage, Delphi items that complemented one another and served common learning goals were clustered in a manner akin to content analysis and grouped under “thematic units.” Accordingly, each thematic unit was conceptualized as a coherent content structure comprising multiple Delphi items that collectively represent the targeted learning outcomes within a given domain. All thematic domains, Self-Awareness and Identity, Emotional Intelligence and Empathy, Future Readiness, Well-Being and Mindfulness, Thinking Skills and Problem Solving, Physical Development and Movement, Language and Culture, Community and Collaboration, Philosophy, Nature, Agriculture, and Ecological Awareness, Arts and Creativity, Technology and Artificial Intelligence, and Science and Quantum Concepts, were represented in the final list. The Table 4 reports, for each thematic unit, the mean expert ratings of the items included, the average standard deviation (SD) associated with these learning outcomes, the percentage of experts assigning the maximum score, and the average coefficient of variation (CV) calculated at the thematic-unit level. In this context, the reported mean values do not represent the psychometric averages of individual learning outcomes; rather, they reflect descriptive and thematic summaries of expert evaluations for the set of outcomes clustered under each thematic unit. The findings indicate that, by the end of the third round, the mean scores of items grouped under the thematic units were consistently high (M ≈ 4.6). This suggests that experts evaluated the vast majority of the proposed learning outcomes within the WAY curriculum as *highly appropriate*. The low standard deviation (SD) values associated with these outcomes indicate that expert ratings converged closely, reflecting a homogeneous distribution of opinions. Furthermore, it was determined that for all learning outcomes, at least 75% of the experts assigned the maximum score. No items falling below this threshold were included in the final list. To further quantify the level of consensus among expert judgments, coefficients of variation (CV) were calculated. The results indicate a strong degree of agreement, as the average CV values for the learning outcomes within thematic units ranged between 0.08 and 0.14 in the third round, representing very low variability. In Delphi studies, CV values below 0.25 are widely regarded as indicative of strong consensus. In the present study, CV values of ≤ 0.15 were obtained for all thematic units in the final round, demonstrating a high level of consistency in expert judgments (Qin et al., 2024). Accordingly, all learning outcomes retained under the thematic units at the conclusion of the third round were considered important for the WAY curriculum and were approved as statistically consensual content by the expert panel.

**Table 4:** Statistical results of the WAY curriculum.

Theme	Thematic unit	Number of items (n)	M	SD	Full score (%)	CV
Self-awareness and identity	Self and identity	6	4.78	0.36	92	0.08
	Awareness of emotions	5	4.74	0.38	90	0.08
	Recognizing and interpreting emotions	3	4.85	0.31	95	0.06
	Strengths and potentials	3	4.72	0.40	89	0.09
	Personal boundaries and needs	4	4.68	0.42	87	0.09
	Intrinsic motivation and purpose	3	4.80	0.33	94	0.07
	Construction of self-concept	3	4.66	0.45	86	0.10
	Self-compassion and self-respect	3	4.82	0.34	93	0.07
Emotional intelligence and empathy	Emotional awareness	4	4.79	0.35	93	0.07
	Understanding others’ emotions	4	4.76	0.37	91	0.08
	Empathy skills	4	4.83	0.32	94	0.07
	Expression of emotions	4	4.71	0.41	88	0.09
	Regulation of emotional responses	3	4.65	0.44	85	0.10
	Social sensitivity	3	4.69	0.42	86	0.09
	Empathy-based conflict approaches	3	4.74	0.39	90	0.08
Future readiness	Concept of the future and time perception	5	4.63	0.46	84	0.10
	Change, uncertainty, and adaptation	4	4.68	0.43	86	0.09
	Future skills	3	4.81	0.34	94	0.07
	Life goals and orientation	3	4.72	0.40	89	0.08
	Responsibility and initiative	3	4.77	0.36	92	0.08
	Positioning the self toward the future	3	4.66	0.45	86	0.10
Well-being and mindfulness	Foundations of well-being	4	4.82	0.33	95	0.07
	Psychological resilience	4	4.78	0.36	93	0.08
	Emotional balance	3	4.71	0.41	88	0.09
	Stress and challenges	3	4.67	0.44	86	0.09
	Coping strategies	4	4.76	0.37	92	0.08
	Present-moment awareness and mindfulness	4	4.85	0.30	96	0.06
	Self-care and healthy habits	3	4.73	0.39	90	0.08
Thinking skills and problem solving	Nature of thinking	4	4.60	0.48	82	0.10
	Critical thinking	2	4.72	0.55	85	0.12
	Analytical and logical reasoning	3	4.68	0.44	87	0.09
	Identifying problem situations	4	4.64	0.46	84	0.10
	Problem-solving processes	3	4.70	0.42	88	0.09
	Generating alternative solutions	4	4.66	0.45	86	0.10
	Decision-making and evaluation of outcomes	3	4.62	0.49	83	0.11
Physical development and movement	Body–mind relationship	4	4.65	0.45	85	0.10
	Bodily awareness	3	4.60	0.48	83	0.10
	Movement and emotions	3	4.58	0.50	82	0.11
	Self-regulation through movement	2	4.62	0.55	84	0.12
	Breathing, relaxation, and rhythm	3	4.70	0.42	88	0.09
	Physical activity and well-being	4	4.74	0.38	90	0.08
Language and culture	Language and meaning	3	4.62	0.45	84	0.10
	Oral expression	4	4.70	0.41	88	0.09
	Written expression	5	4.78	0.36	92	0.08
	Storytelling, narrative, and text	4	4.74	0.38	90	0.08
	Cultural heritage	5	4.80	0.34	94	0.07
	Cultural diversity	3	4.68	0.43	86	0.09
	Multicultural perspectives	3	4.72	0.40	89	0.08
Community and collaboration	Community and belonging	4	4.76	0.37	92	0.08
	Culture of collaboration	4	4.79	0.35	93	0.07
	Teamwork	5	4.83	0.32	95	0.07
	Social responsibility	3	4.70	0.42	88	0.09
	Democratic participation	4	4.68	0.44	86	0.09
	Shared goals and cooperation	5	4.81	0.33	94	0.07
Philosophy	Inquiry and thinking	5	4.72	0.40	89	0.08
	Meaning and existence	4	4.66	0.44	86	0.09
	Values and ethics	5	4.78	0.36	92	0.08
	Moral dilemmas	3	4.60	0.50	82	0.11
	Critical philosophical approaches	4	4.68	0.43	86	0.09
	Diverse systems of thought	3	4.64	0.47	84	0.10
Nature, agriculture, and ecological awareness	Relationship with nature	4	4.60	0.48	83	0.10
	Ecological systems	3	4.55	0.52	80	0.11
	Human–nature interaction	4	4.62	0.46	84	0.10
	Sustainable living	4	4.68	0.44	86	0.09
	Agriculture and food cycles	4	4.58	0.50	82	0.11
	Ecological responsibility	4	4.72	0.42	88	0.09
Arts and creativity	Foundations of creativity	3	4.75	0.38	91	0.08
	Creative thinking	4	4.82	0.33	95	0.07
	Artistic expression	4	4.78	0.36	92	0.08
	Aesthetics and sensitivity	3	4.70	0.42	88	0.09
	Original product design	5	4.85	0.30	96	0.06
	Interdisciplinary arts	3	4.72	0.40	89	0.08
Technology and artificial intelligence	Technology and humanity	3	4.62	0.46	84	0.10
	Digital literacy	4	4.70	0.41	88	0.09
	Concept of artificial intelligence	3	4.68	0.43	86	0.09
	Societal impacts of artificial intelligence	5	4.76	0.37	92	0.08
	Ethical and safe use of technology	4	4.72	0.39	90	0.08
	Problem solving through technology	5	4.78	0.36	93	0.08
Science and quantum concepts	Scientific curiosity	4	4.68	0.43	86	0.09
	Scientific thinking	5	4.75	0.38	91	0.08
	Causality and systems	4	4.62	0.46	84	0.10
	Probability and uncertainty	4	4.60	0.48	83	0.10
	Quantum thinking	4	4.58	0.50	82	0.11
	Philosophy of science	3	4.64	0.47	84	0.09

*M* = mean; *SD* = standard deviation; *CV* = coefficient of variation.

The 312 learning outcomes are organized hierarchically across four developmental bands corresponding to the Turkish national education stages: early childhood (ages 4–6), primary education (grades 1–4), lower secondary (grades 5–8), and upper secondary (grades 9–12). Within each thematic domain, outcomes were sequenced in alignment with developmental progression principles: foundational awareness and self-referential competencies are emphasized in early childhood units, while more complex, reflective, and socially oriented outcomes are concentrated in secondary levels. This developmental scaffolding was informed by established frameworks such as CASEL’s developmental benchmarks and Vygotsky (1978) zone of proximal development. To illustrate the developmental scaffolding of the finalized framework, Appendix 1 presents the distribution of learning outcomes across four national education stages for each thematic domain, along with representative competency descriptors.

## Conclusion and discussion

This study presents the WAY curriculum as a social, emotional, and holistic educational framework designed to support learners’ cognitive, social, emotional, and identity-related development by integrating self-awareness, emotional regulation, social interaction, meaning-making, and identity development within a coherent curricular structure. By combining expert consensus with theoretically grounded themes, the study moves beyond a purely technical item-validation exercise and offers a broad curriculum framework aligned with contemporary educational perspectives on socio-emotional learning, ethical engagement, and holistic development. Importantly, these findings should be interpreted as supporting the expert validation of a learning outcome framework, rather than as evidence of the implementation or effectiveness of a fully developed curriculum.

The present study demonstrated a notably high level of participation and commitment among the Delphi panelists. The fact that 72.8% of the invited experts participated in the first round, and that retention rates across subsequent rounds remained well above the minimum threshold of 70%, indicates that the consensus reached regarding the WAY curriculum is methodologically robust (Sumsion, 1998). Moreover, a substantial proportion of experts provided qualitative feedback alongside their quantitative ratings in each round, thereby enriching the dataset. These findings indicate sustained expert engagement across rounds and suggest that the consensus process benefited from both stable participation and substantive qualitative input (Hsu and Sandford, 2007).

At the same time, the present study should be interpreted as developing a consensus-based curriculum framework rather than a fully implementable curriculum design. Although the Delphi process yielded 312 endorsed learning outcomes organized into 84 thematic units, a complete curriculum also requires age-sensitive sequencing, pedagogical models, instructional materials, assessment strategies, and implementation planning. Accordingly, the present article represents a foundational blueprint for curriculum construction rather than the final pedagogical product itself.

In the present study, the level of expertise among panelists and the credibility of their judgments were further supported by quantitative indicators. Across the three Delphi rounds, the panel’s mean authority coefficient values indicated a high level of subject-matter competence among experts across the relevant thematic domains (Giannarou and Zervas, 2014). These consistently high authority coefficients support the credibility of the expert judgments obtained in the study. In parallel, the increasing consistency of experts’ importance ratings across successive rounds reflects a progressive strengthening of consensus. The steady rise in Kendall’s W coefficients calculated after each round indicates that agreement among panelists gradually intensified. While complete consensus was not evident at the outset, given the interdisciplinary composition of the panel, anonymous feedback and repeated rounds of evaluation substantially reduced divergences in expert opinions by the third round. In other words, despite the diversity of disciplinary backgrounds represented, the panel demonstrated substantial convergence by the final round. Ultimately, the WAY curriculum emerged as an expert-informed framework comprising 13 thematic domains, 84 units, and 312 learning outcomes, collectively endorsed through expert consensus. This outcome indicates that the content of the WAY curriculum was broadly accepted by a diverse group of experts and received broad interdisciplinary endorsement.

The literature suggests that holistic curricula such as WAY contribute to individuals’ development across multiple dimensions. Bruner (1996) emphasized that effective educational programs are engaging, socially grounded, and collaborative, and that learning should be conceptualized not as the transmission of information but as a process of meaning-making. Similarly, Vygotsky’s (1978) sociocultural learning theory posits that learning develops through interaction with others and that cognitive and social skills are enhanced through collaboration. The Community and Collaboration theme embedded in the WAY curriculum, which promotes learning through interaction with peers and adult facilitators, aligns closely with Vygotsky’s theoretical framework. In addition, Lipman’s (2003) *philosophy for children* approach has demonstrated that inquiry-based dialogue and storytelling foster children’s critical and creative thinking skills while enhancing empathy and social awareness. In this regard, the WAY curriculum may support students’ processes of meaning-making and value construction through narratives, stories, and philosophical discussions. The inclusion of nature- and arts-based activities in the WAY curriculum is also grounded in well-established findings in educational research. Louv (2005) argued that the growing disconnection of children from nature can result in what he termed “nature-deficit disorder,” whereas regular engagement with natural environments has been shown to enhance children’s well-being, creativity, and overall health. Children who engage in play in natural settings are more likely to invent their own games, exercise imagination, and collaborate with others, and they tend to demonstrate stronger performance in science-related domains. Accordingly, the WAY curriculum enriches learning by extending educational experiences beyond the classroom through activities such as nature observation, gardening, and agricultural practices. From the perspective of arts education, Eisner (2002) highlighted the educational value of the arts in cultivating complex forms of thinking that enable students to cope with uncertainty. The inclusion of music, visual arts, and drama within the WAY curriculum may therefore foster students’ expressive capacities and creative thinking skills.

Finally, the development of thinking skills has become an important objective in contemporary education systems. Wing (2006) underscored that thinking skills, particularly those related to problem solving and system design, constitute essential competencies for all learners and should be cultivated from an early age. Moreover, with the widespread integration of artificial intelligence tools into everyday life, students are increasingly expected not only to use technology effectively but also to evaluate it critically and ethically (García-López and Trujillo-Liñán, 2025). The WAY curriculum addresses these demands by incorporating learning outcomes related to coding, robotics, digital citizenship, and artificial intelligence applications, thereby equipping students with competencies necessary for the technological landscapes of the future. At the same time, it supports the development of ethical awareness and human values across all domains of life, including digital environments. Taken together, the theoretical literature and the expert consensus reported in this study suggest that the WAY curriculum adopts a holistic approach to education by integrating cognitive, affective, social, and ethical dimensions of development. Taken together, this theoretical and empirical body of evidence suggests that the WAY curriculum adopts a holistic approach to education by integrating cognitive, affective, social, and ethical dimensions of development. The WAY curriculum may therefore be characterized as a contemporary, multi-layered educational framework intended to support competencies considered relevant for future educational contexts.

## Implications

The findings of this study have several implications for the field of educational sciences. The framework developed through the WAY curriculum may be considered a structured example of a holistic educational approach that brings together cognitive, affective, social, and identity-related dimensions of learning within a common curricular perspective. Holistic education is generally concerned with supporting learners’ development across multiple interrelated domains, including cognitive, emotional, social, physical, creative, and ethical dimensions (Miller, 2007). In this respect, the WAY framework may be seen as an attempt to translate such a perspective into a curriculum-oriented structure by bringing together developmental domains such as self-awareness, self-regulation, social awareness, creativity, ecological sensitivity, technological competence, and identity-related growth within a common framework.

The expert consensus obtained in this study is broadly consistent with long-standing theoretical arguments that education should extend beyond approaches that prioritize academic knowledge alone. In particular, the thematic structure of the WAY curriculum brings together social–emotional learning competencies with widely discussed 21st-century skills, including creativity, critical thinking, collaboration, and digital literacy. The convergence of expert judgments across diverse disciplinary backgrounds may therefore be interpreted as support for the view that contemporary curricula can benefit from more integrated and transdisciplinary approaches to learner development (Voogt and Roblin, 2012; Miseliunaite et al., 2022). At the same time, the inclusion of identity-related dimensions alongside self-awareness, social–emotional competencies, ecological awareness, and technological literacy suggests that curriculum design may also provide space for learners to engage with questions of self-understanding, personal meaning, and their relationship to others and the wider world.

Another noteworthy implication concerns the coexistence of themes related to nature, ecology, technology, and artificial intelligence within the same curriculum framework. Rather than treating these as separate or competing areas, the WAY framework brings them into relation with social–emotional and identity-related development. In this sense, the framework may offer a useful point of reference for future curriculum work seeking to connect sustainability awareness, digital literacy, and personal-social development within a broader educational perspective. Overall, the WAY curriculum may be regarded as a broad, expert-informed framework that could inform future efforts in holistic curriculum development and interdisciplinary educational design.

## Limitations

Several limitations should be acknowledged when interpreting the findings of this study. First, the study relied on the Delphi technique, which is inherently based on expert judgment. Although this approach is well suited to the development of a consensus-based curriculum framework, it does not provide empirical evidence regarding how the proposed learning outcomes function in authentic educational settings. Thus, the findings should be interpreted as indicating expert endorsement, theoretical coherence, and perceived curricular relevance rather than demonstrated instructional effectiveness. The practical value, feasibility, and student-level impact of the WAY framework remain to be examined through empirical implementation studies.

Second, the consensus-oriented structure of the Delphi technique may have reduced the visibility of minority or unconventional perspectives. Although panelists responded anonymously, the provision of aggregated group feedback in later rounds may have encouraged some participants to move toward the central tendency. While such convergence is expected in Delphi studies and supports the refinement of shared priorities, it may also result in the exclusion of ideas that are less widely endorsed but still potentially meaningful in specific educational contexts. In the present study, some items were removed after failing to achieve sufficient support, even though they may retain contextual, emergent, or future relevance for particular settings. For this reason, the final framework should not be interpreted as exhausting all potentially valuable curricular possibilities, but rather as representing the set of learning outcomes that achieved sufficient expert support within the present consensus process.

Third, the expert panel was drawn entirely from Türkiye. Although participants represented all seven geographical regions of the country, the resulting framework remains situated within the Turkish educational context. This strengthens contextual relevance for national curriculum discussions, but limits direct cross-cultural generalizability. Holistic education priorities, identity-related development, and the relative importance assigned to social–emotional, ecological, and technological competencies may vary across cultural and institutional settings. Accordingly, the transferability of the WAY framework to other systems should be approached with caution until it is examined through broader international and comparative validation efforts.

Fourth, the professional composition of the panel may also have influenced the direction of the consensus. The predominance of early- and mid-career participants may have favored more contemporary or progressive orientations toward curriculum design while comparatively limiting the influence of senior practitioners with longer-term implementation experience. Although the interdisciplinary breadth of the panel was a methodological strength, differences in disciplinary density across thematic areas may also have shaped patterns of consensus in subtle ways. These features do not invalidate the framework, but they do suggest that the final structure reflects a particular expert configuration rather than a universally settled curricular model.

Finally, the WAY framework developed in this study should be understood as a first-stage curriculum blueprint rather than a fully implementable curriculum. While the study generated 312 expert-endorsed learning outcomes organized into 84 thematic units under 13 domains, a complete curriculum design requires further work on developmental sequencing, instructional planning, assessment tools, teacher guidance, and implementation supports. This is especially important for ensuring applicability across diverse school environments, including under-resourced settings where implementation demands may be more pronounced. Therefore, claims regarding the framework’s feasibility should be interpreted at the conceptual and content level, not as evidence of classroom readiness or demonstrated educational effectiveness.

Future research should move beyond expert validation toward staged empirical implementation. Small-scale pilot studies, design-based research, and mixed-methods classroom applications may help examine the usability, developmental appropriateness, and contextual adaptability of the framework. Subsequent studies may also evaluate its influence on students’ social–emotional, cognitive, identity-related, and well-being outcomes through quasi-experimental and longitudinal designs conducted across diverse educational settings.

## Data Availability

The raw data supporting the conclusions of this article will be made available by the authors, without undue reservation.
