# Supplementary material for: “Who Are You”: development of a holistic, social-emotionally grounded curriculum framework using the Delphi technique
Source: Front Psychol. 2026 May 11;17:1776122. doi: 10.3389/fpsyg.2026.1776122 (PMC13200526; doi:10.3389/fpsyg.2026.1776122)
Supplement: Supplementary file 1 [file Supplementary_file_1.DOCX]

### ****Appendix 1. WAY Curriculum: Developmental Level × Thematic Domain Matrix****

| **Thematic Domain** | **Units (n)** | **Items (n)** | **Developmental Focus** | **Early Childhood (Ages 4–6)** | **Primary Education (Grades 1–4)** | **Lower Secondary (Grades 5–8)** | **Upper Secondary (Grades 9–12)** |
| --- | --- | --- | --- | --- | --- | --- | --- |
|  |  |  |  | **No. of outcomes / Representative competency** | **No. of outcomes / Representative competency** | **No. of outcomes / Representative competency** | **No. of outcomes / Representative competency** |
| **Self-Awareness & Identity** | **8** | **30** | Foundational self-recognition & emotion naming | **(6 outcomes)**  Name basic emotions; recognize personal likes/dislikes | **(8 outcomes)**  Identify personal strengths; express needs and boundaries | **(9 outcomes)**  Develop self-concept; practice self-compassion and motivation | **(7 outcomes)**  Integrate identity; manage identity conflicts; articulate values |
| **Emotional Intelligence & Empathy** | **7** | **25** | Emotion recognition → Empathy → Regulation | **(5 outcomes)**  Recognize own emotions; basic empathy toward peers | **(7 outcomes)**  Understand others' emotions; express emotions constructively | **(7 outcomes)**  Regulate emotional responses; empathy-based conflict resolution | **(6 outcomes)**  Advanced social sensitivity; nuanced empathic reasoning |
| **Future Readiness** | **6** | **21** | Time perception → Goal-setting → Adaptive agency | **(3 outcomes)**  Distinguish past/present/future; simple goal-setting | **(5 outcomes)**  Plan short-term goals; understand change and adaptation | **(7 outcomes)**  Identify future skills; develop life orientation and initiative | **(6 outcomes)**  Position self toward an uncertain future; strategic life planning |
| **Well-Being & Mindfulness** | **7** | **27** | Physical & psychological well-being through all stages | **(6 outcomes)**  Identify feelings of calm/stress; basic self-care routines | **(7 outcomes)**  Practice mindfulness; healthy habits; simple coping strategies | **(7 outcomes)**  Build psychological resilience; manage stress and challenges | **(7 outcomes)**  Emotional balance; advanced mindfulness; well-being advocacy |
| **Thinking Skills & Problem Solving** | **7** | **23** | Concrete → Analytical → Critical → Systems thinking | **(3 outcomes)**  Explore cause-effect; ask 'why' questions about the world | **(6 outcomes)**  Identify problem situations; generate simple solutions | **(7 outcomes)**  Critical and analytical reasoning; evaluate alternative solutions | **(7 outcomes)**  Systems thinking; responsible decision-making; evaluate outcomes |
| **Physical Development & Movement** | **6** | **19** | Bodily awareness & movement across developmental stages | **(5 outcomes)**  Gross motor play; body-part awareness; breathing rhythms | **(5 outcomes)**  Coordinated movement; link movement to emotions | **(5 outcomes)**  Self-regulation through movement; physical activity habits | **(4 outcomes)**  Mind-body integration; lifelong physical well-being |
| **Language & Culture** | **7** | **27** | Expression, narrative, and cultural identity | **(5 outcomes)**  Oral storytelling; explore language through play and song | **(7 outcomes)**  Oral & written expression; cultural heritage appreciation | **(8 outcomes)**  Narrative and text literacy; multicultural perspectives | **(7 outcomes)**  Critical language use; intercultural communication; cultural diversity |
| **Community & Collaboration** | **6** | **25** | Belonging → Cooperation → Social responsibility | **(5 outcomes)**  Sense of belonging; basic turn-taking and sharing | **(6 outcomes)**  Teamwork skills; community and collaboration culture | **(7 outcomes)**  Shared goals; social responsibility; democratic participation | **(7 outcomes)**  Civic engagement; collaborative leadership; cooperative ethics |
| **Philosophy** | **6** | **24** | Inquiry & meaning-making from simple to complex | **(3 outcomes)**  Wonder and curiosity; simple 'what is fair?' discussions | **(6 outcomes)**  Inquiry and thinking skills; explore meaning and existence | **(8 outcomes)**  Moral dilemmas; values and ethics; diverse systems of thought | **(7 outcomes)**  Critical philosophical approaches; ethics in modern contexts |
| **Nature, Agriculture & Ecological Awareness** | **6** | **23** | Nature connection → Ecological systems → Sustainability | **(5 outcomes)**  Explore natural environments; observe plants/animals | **(6 outcomes)**  Ecological systems; human-nature interaction; food cycles | **(6 outcomes)**  Sustainable living practices; ecological responsibility | **(6 outcomes)**  Environmental advocacy; systems-level ecological thinking |
| **Arts & Creativity** | **6** | **24** | Creative expression → Aesthetic sensibility → Original design | **(5 outcomes)**  Free creative play; explore art materials and music | **(6 outcomes)**  Artistic expression; foundations of creativity | **(7 outcomes)**  Aesthetics and sensitivity; creative thinking processes | **(6 outcomes)**  Original product design; interdisciplinary arts integration |
| **Technology & Artificial Intelligence** | **6** | **23** | Digital literacy → AI literacy → Ethical technology use | **(3 outcomes)**  Safe and supervised digital exploration; technology as a tool | **(5 outcomes)**  Digital literacy basics; technology and humanity relationship | **(8 outcomes)**  Concept of AI; societal impacts; ethical and safe technology use | **(7 outcomes)**  AI applications; problem-solving through technology; digital ethics |
| **Science & Quantum Concepts** | **6** | **21** | Scientific curiosity → Causal reasoning → Quantum thinking | **(3 outcomes)**  Observe and ask questions; basic science curiosity | **(5 outcomes)**  Scientific thinking; causality and simple experiments | **(6 outcomes)**  Probability and uncertainty; systems thinking | **(6 outcomes)**  Quantum thinking; philosophy of science; complex systems |
| **TOTAL** | **84** | **312** | **13 Thematic Domains** | **57** | **79** | **92** | **83** |

***Note.*** *The number of outcomes per developmental level reflects the relative conceptual emphasis within each thematic domain across the four Turkish national education stages. Learning outcomes were distributed in accordance with developmental progression principles (CASEL, 2023; Vygotsky, 1978). EC = Early Childhood (ages 4–6); PS = Primary Education (Grades 1–4); LS = Lower Secondary Education (Grades 5–8); US = Upper Secondary Education (Grades 9–12). Total items (N = 312) represent the final consensual item pool following three Delphi rounds (Kendall's W = 0.83, p < .001).*
